# An Introduction to Pathology and Morbid Anatomy

**Published:** 1896-03

**Authors:** 


An Introduction to Pathology and Morbid Anatomy. By T. Henry Green, M. D., F. R. C. P., Physician and Special Lecturer on Clinical Medicine at Charing Cross Hospital, and Physician to the Hospital for Consumption and Diseases of the Chest, Brompton. Seventh American from the eighth English edition. Revised and enlarged by H. Montague Murray, M. D., F. R. C. P. Illustrated by 224 engravings; pp. 598. Philadelphia: Lea Brothers & Co. 1895.

In the November, 1889, number of the Journal, p. 251, was reviewed at some length the sixth American edition of this favorite work and its salient points commented on. No such review is necessary for this volume, as the changes are few and the increase in size is due mainly to the addition of new illustrations. Some chapters have been remodeled and some entirely rewritten, especially the chapter on diseases of the nervous system. This has been contributed by Dr. Mott and although concise and abbreviated is a good review of the pathological findings of the cord and brain. Perhaps not enough attention has been given to the neuron theory and its change in disease, but the author pleads lack of space. The engravings are excellent and are nearly all photographed from the writer's own specimens.

The chapter on vegetable parasites, consisting of nearly 100 pages, includes the latest additions to our knowledge of germ-life.

The chapters on tumors and inflammations are in the main similar to those in the preceding edition with few additions. The author belongs as yet to the followers of Metchnikoff and the phagocytic theory and reviews this writer's work very clearly.

The enlargement of the volume will tend somewhat to keep it from the medical student, but will make it more acceptable to the profession at large.

The typographical work is on a par with the high plane attained by this favorite house. W. C. K.

